# Peripheral oxygen saturation levels as a guide to avoid hyperoxia: an observational study

**DOI:** 10.1186/s13049-025-01323-4

**Published:** 2025-01-15

**Authors:** Renate Stolmeijer, Jan C. ter Maaten, Jack Ligtenberg, Ewoud ter Avest

**Affiliations:** 1 Department of Acute Care, University Medical Centre Groningen, Groningen, the Netherlands; 2 Department of Internal Medicine, University Medical Centre Groningen, Groningen, The Netherlands; 3 London’s Air ambulance, London, UK

**Keywords:** Hyperoxia, Pre-hospital, Oxygen, P/F ratio

## Abstract

**Background:**

As iatrogenic hyperoxia has been related to adverse outcomes in critically ill patients, guidelines advise to titrate oxygen to physiological levels. In the prehospital setting where partial arterial oxygen (PaO_2_) values are often not readily available, titration of oxygen is based on peripheral oxygen saturations (SpO2). In this study we aimed to investigate the efficacy of SpO_2_ guided oxygen titration in the prevention of hyperoxia.

**Methods:**

In a retrospective observational cohort study of patients included in the Acutelines data- and biobank of the University Medical Center Groningen between September 2020 and March 2023, we collected blood gas samples and triage data of sequentially included patients who received oxygen at the moment they were presented in the emergency department (ED). PaO_2_ values were compared to (concurrently measured) SpO_2_ values, and to patient- and treatment characteristics and P/F ratios were calculated in order to investigate the efficacy of SpO_2_ based oxygen titration for various subgroups.

**Results:**

Blood gas samples were obtained for 1042 patients, of which 178 (17.1%) had hyperoxia (PaO_2_ levels > 13.5 kPa). SpO_2_ readings were available for 170 of these, 68 of which (40%) had SpO_2_ values above the recommended target range (94–98%; 88–92% for patients with COPD) whereas 102 patients (60%) had SpO2 values within- or even below the recommended target range. Many of these patients (44.1%) received oxygen through a low-flow device (nasal canula), and these patients almost invariably (84.4%) were *not* compromised in their ventilation (P/F ratio’s > 300).

**Conclusion:**

When oxygen is titrated based on SpO_2_ levels, this results in hyperoxemia in a significant proportion of the patients. Health care providers should especially be reluctant to administer (low flow) oxygen as a standard of care to patients who do not have clear respiratory compromise, as these patients are at a high risk of developing (occult) hyperoxia.

## Background

Acutely ill patients often have an increased oxygen demand, a compromised oxygen uptake- or transport or both [1]. Consequently guidelines historically recommended the pragmatic suppletion of oxygen with a high inspired oxygen fraction (FiO_2_) in these patients in order to avoid hypoxia [2, 3]. 

However, over the past decade it has become obvious that (too) generous suppletion of oxygen is not without risk. Prolonged episodes of hyperoxia have been found to be associated with a diminished self-reliance and a higher mortality [4–7]. This is likely mediated by reactive oxygen species (ROS), formed when excess oxygen is administered [8–10]. These ROS affect systemic- and pulmonary vascular tone, resulting in an unwanted decrease in cardiac output (CO) [4]. Further, ROS can damage cellular structures, especially mitochondria, eventually resulting in permanent endothelial damage. Recognition of these deleterious effects has resulted in guideline changes, and several guidelines now recommend to titrate oxygen to physiological levels [11, 12]. 

This is often done by measuring partial arterial oxygen pressures (PaO_2_) obtained through arterial blood gas analysis (ABGA). However, ABGA is not always available (as in the prehospital setting) or sometimes undesirable (as in the ED): Although ABGA is integral to assessing emergency department (ED) patients with acute respiratory or metabolic disease, it is a painful procedure with potential complications and therefore not standard of care to all patient in the ED [13]. In these situations, oxygen is administered based on peripheral capillary oxygen saturation (SpO_2_) values measured by finger plethysmography. Several guidelines recommend to aim for an SpO_2_ of 94–98% (or an SpO_2_ of 88–92% in patients with chronic obstructive pulmonary disease (COPD) GOLD III or IV) [11, 12]. Titration of oxygen based on SpO_2_ readings however, requires a reliable and stable SpO_2_ trace, which may not always be available, especially when patients have dysrhythmia’s, when they are in shock, or when there are movement artefacts (as in the prehospital setting) [14]. This may affect the efficacy of SpO_2_ guided oxygen administration.

Therefore, in the present study, we set out to investigate the efficacy of SpO_2_ guided oxygen titration in the prevention of hyperoxia, and we tried to identify modifiable patient- or treatment characteristics associated with hyperoxia.

## Methods

### Study setting and design

A retrospective observational cohort study was performed of patients who presented in the ED of the University Medical Center Groningen (UMCG), a tertiary care facility in the northern part of the Netherlands with approximately 25.000 ED visits each year, and who were included in the Acutelines data-and biobank at the time of their visit Ethical approval was obtained from the Central ethical Committee (CTc) of the UMCG), protocol number 11120.

### Study population

Patients were included in the present study when they met the following criteria:

- Oxygen was administered at the time of presentation/triage in the ED.
- An arterial blood gas sample was performed at triage and showed hyperoxemia (PaO2 > 13.5 kPa).
- Patients had consented to participate in the Acutelines data- and biobank during their ED visit.

Patients were excluded when no plethysmographic SpO_2_ readings were obtained or recorded at the time of their presentation.

### Data acquisition

Data for this cohort study were obtained from the Acutelines data- and biobank [15]. Acutelines is a multidisciplinary prospective hospital-based cohort study in the ED of the UMCG. Acutelines is approved by the medical ethics board of the UMCG and registered under trial registration number NCT04615065 at ClinicalTrials.gov. The cohort population is broadly representative of the population with acute medical conditions in the northern part of the Netherlands. Primary screening of patients for eligibility on arrival at the ED is performed 24 h a day by the ED nurse together with a trained research team. Participants are asked to give written informed consent, with a proxy if necessary. Data were collected and managed using REDCap (Vanderbilt University, Nashville, TN, USA) electronic data capture tools hosted at the UMCG [16, 17]. Bedside monitoring data were automatically captured and stored, and information from other data sources including the electronic health records of the hospital was imported via the electronic patient file (EPIC systems, Boston, MA, USA).

The following variables were collected for all included patients: Demographic variables, relevant (cardio-pulmonary) past medical history, smoking status, triage category (urgency) according to the Netherlands Triage Standard (NTS), the full set of vital signs obtained during ED triage, and the concentration of oxygen administered (FiO_2_). FiO_2_ was estimated from the method of oxygen delivery registered, and the registered oxygen flow (in case of a nasal cannula) [18]. The ratio’s between PaO_2_ and FiO_2_ (P/F ratio) were calculated from the first ABGA results after triage to objectify presence- and severity of acute respiratory distress syndrome (ARDS). A P/F ratio < 300 was regarded as indicative of ARDS [19]. 

### Outcomes

Primary outcome was the incidence of occult hyperoxia* in patients receiving oxygen titrated based on SpO_2_.

Secondary endpoints were patient-or treatment factors associated with the occurrence of hyperoxia.

* Occult hyperoxia was defined as hyperoxia (PaO2 > 13.5) with an SpO2 below the target range of 94–98% (or 88–92% in patients with COPD GOLD III-IV).

### Sample size

As the primary purpose of this pilot study was not hypothesis testing, no formal sample size calculation was made [20]. We aimed to include at least 100 patients.

### Statistical analysis

Results are displayed in numbers, percentages and averages. Differences regarding secondary endpoints between identified subgroups where tested using Chi [2] test, Kruskal Wallis test or independent sample t-tests where appropriate. Bonferroni adjustments were applied to correct for multiple comparisons between subgroups of patients with obvious- and occult hyperoxemia, with a *p* < 0.005 being considered statistically significant. All statistical analysis was done with SPSS 23.0 (SPSS Inc, Chicago, Illinois, USA).

## Results

In total, 1042 patients met eligibility criteria during the study period. Normoxia- or hypoxia was present in 864 patients whereas hyperoxia was present in 178 patients (17,1%). For 8 patients with confirmed hyperoxia no SpO_2_ values were registered during triage. Further results refer to the remaining 170 patients.

### Patient characteristics

The majority of the included patients (57.6%) was male. Mean age at the time of presentation was 62 years. Most included patients visited the ED with complaints attributable to sepsis (47%). About a third of the patients had an underlying chronic lung condition. Oxygen was delivered by nasal cannula (*n* = 74), venturi mask (*n* = 11), non-rebreathing mask (*n* = 59), or by (non-invasive) assisted ventilation (*n* = 26) (Table 1).

**Table 1:** Patient characteristics of patients (*n* = 170) presented in the ED with hyperoxia (PaO_2_ > 13,5 kPa) in their arterial blood gas analysis, stratified by SpO_2_ recorded by finger plethysmogram at presentation

	Total *N* = 170	Occult hyperoxia (SpO_2_ ≤ target range*, *n* = 102)	Obvious hyperoxia SpO_2_ > target range*, *n* = 68)	*p*-value
Gender				0.800
- Male - Female	98 (57.6) 72 (42.4)	58 (56.9) 44 (43.1)	40 (58.8) 28 (41.2)	
Age	62.3 (15.8)	63.9 (16.4)	59.7 (14.5)	0.278
O2 delivery device				0.458
- Nasal canula - Venturi mask - Non rebreathing mask - Non invasive ventilation - Tube or supraglottic airway device	74 (43.5) 11 (6.5) 59 (34.7) 7 (4.1) 19 (11.2)	45 (44.1) 6 (5.9) 39 (38.2) 4 (3.9) 8 (7.8)	29 (42.6) 5 (7.4) 20 (29.4) 3 (4.4) 11 (16.2)	
Triage urgency colour code				0.227
- Yellow (urgent) - Orange (emergent) - Red (immediate) - Missing	55 (32.4) 88 (51.8) 23 (13.5) 4 (2.4)	35 (34.3) 54 (52.9) 10 (9.8) 3 (2.9)	20 (29.4) 34 (50) 13 (19.1) 1 (1.5)	
Smoker	45 (26.5)	24 (23.5)	21 (30.9)	0.042
Comorbidities**				
- Cardiac - Pulmonary	29 (17) 54 (31.8)	24 (23,5) 27 (26.5)	5 (7.4) 27 (39.7)	0.006 0.069
Suspected diagnosis in ED				
- Sepsis - COPD exacerbation - Other****	80 (47,1) 12 (7,1) 81 (47,6)	52 (51.0) 3 (2.9) 47 (46.1)	28 (41.2) 9 (13.2) 34 (50)	0.210 0.010 0.616

Legend Table 1. Age is represented as mean (SD), all other values are n (%). Abbreviations: SaO2, peripheral oxygen saturation; O2, oxygen; PaO2, partial arterial oxygen pressure; PCI, percutaneous coronary intervention; CABG, coronary artery bypass grafting; COPD, chronic obstructive lung disease; ED, emergency department. *; SpO_2_ target range is 94–98%, except for patients with COPD GOLD III-IV, in which it is 88–92%. **Co-morbidities: Cardiac comorbidities included heart failure (*n* = 19), myocardial infarction (*n* = 9) PCI or CABG (*n* = 11) or previous heart valve replacement (*n* = 3); Pulmonary comorbidities included COPD GOLD I-II (*n* = 17), COPD GOLD III-IV (*n* = 15) and other chronic pulmonary conditions (*n* = 22). ***; Other diagnoses included: intoxications, anaphylaxis and gastro-intestinal bleed. After Bonferroni adjustment, a *p* value < 0.005 was regarded as significant

### Incidence of hyperoxia

**Fig. 1:**
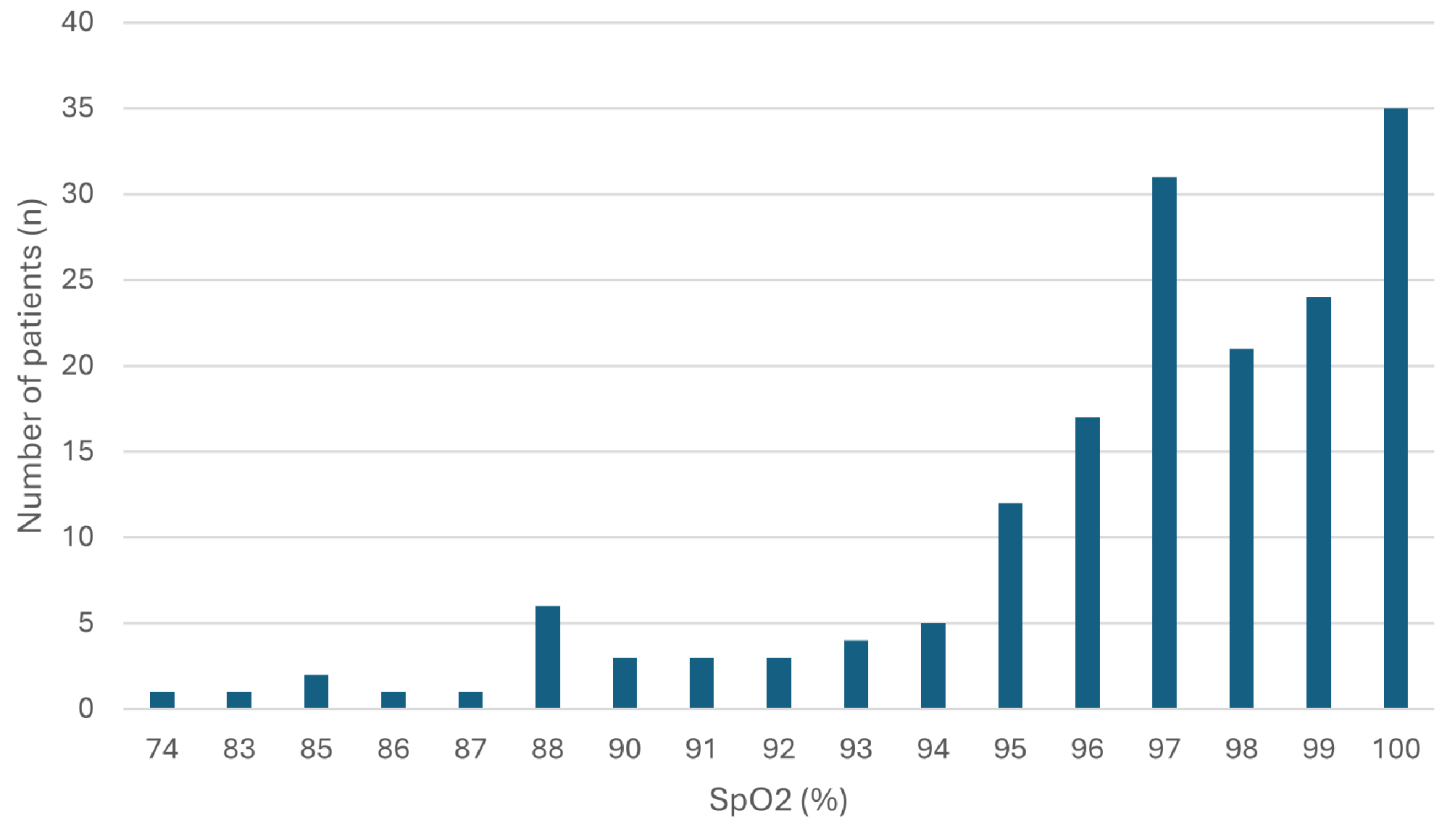
Frequency distribution of SpO_2_ levels of hyperoxic (PaO_2_ > 13,5 kPa patients (n = 170)

Obvious hyperoxia, with SpO_2_ values above the generally recommended target range of 94–98% (88–92% in severe COPD), was present in 68 (40%) patients, whereas occult hyperoxia (PaO2 > 13,5 kPa but with SpO_2_ values within or below the target range) was present in the remaining 102 (60%) of the patients. (Fig. 1).

### Patient and treatment factors associated with hyperoxemia

Stratification of patients based on their SpO_2_ at presentation revealed that patients with occult hyperoxia more often had heart failure or a history of ischemic heart diseases (Table 1), whereas patients with obvious hyperoxia more often had a history of pulmonary diseases (although significance was not reached, Table 1).

Most patients (45/102 (44%)) with occult hyperoxia received oxygen via a nasal canula (low flow oxygen suppletion) (Table 2). These patients were considered less sick (as represented by a lower triage urgency category) than patients receiving high flow oxygen (via NRM or ventimask (VM)) or assisted ventilation). The vast majority of the patients with a nasal canula (84,4%) had a P/F ratio > 300, whereas in patients receiving high-flow oxygen therapy this was the opposite: 75.6% had a P/F ratio < 300. In 4 patients (all receiving oxygen through assisted (non-invasive) ventilation), no P/F ratio could be determined, since no FiO_2_ was registered.

**Table 2:** Patients with occult hyperoxia on presentation in the ED, stratified by method of oxygen suppletion

	Total *n* = 102	Low flow* oxygen suppletion *N* = 45	High flow** oxygen suppletion *N* = 45	Assisted (non-) invasive ventilation *N* = 12	*P*-value
Gender					0.393
- Male - Female	58 (56.9) 44 (43.1)	25 (55.6) 20 (44.4)	24 (53.3) 21 (46.7)	9 (75) 3 (25)	
Age	63.9 (16.4)	64.4 (19.2)	64.0 (14.7)	62.3 (11.3)	0.372
Triage colour					< 0.001
- Yellow - Orange - Red - Missing	35 (34.3) 54 (52.9) 10 (9.8) 3 (2.9)	25 (55.6) 17 (37.8) 2 (4.4) 1 (2.2)	6 (13.3) 33 (73.3) 4 (8.9) 2 (4.4)	4 (33.3) 4 (33.3) 4 (33.3) 0 (0)	
Smoking	24 (23.5)	7 (15.6)	11 (24.4)	6 (50)	0.004
Co-morbidities***					
- Pulmonary - Cardiac	27 (26,5) 24 (23.5)	13 (28,9) 10 (22.2)	9 (20) 10 (22.2)	5 (41,7) 4 (33.3)	0,283 0.695
Suspected ED diagnosis					
- COPDexacerbation - Sepsis - Other****	3 (2.9) 52 (51.0) 47 (46.1)	1 (2.2) 21 (46.7) 23 (51.1)	1 (2.2) 30 (66.7) 14 (31.1)	1 (8.3) 1 (8.3) 10 (83.3)	0.500 0.001 0.004
P/F ratio					< 0.001
- < 300 - > 300 - Missing	47 (46.1) 51 (50) 0 (0)	7 (15.6) 38 (84.4) 0 (0)	34 (75.6) 11 (24.4) 0 (0)	6 (50) 2 (16.7) 4 (33.3)	

Legend Table 2. Age is represented as mean (SD), all other values are n (%). Abbreviations: PCI, percutaneous coronary intervention; CABG, coronary artery bypass grafting; COPD, chronic obstructive lung disease; ED, emergency department; P/F ratio, partial arterial oxygen pressure / inspired oxygen fraction ratio. *Low flow oxygen suppletion is provided by a nasal canula. **High flow oxygen suppletion is provided by a venturi mask or a non-rebreathing mask. ***Co-morbidities: Pulmonary comorbidities included COPD GOLD I-II (*n* = 17), COPD GOLD III-IV (*n* = 15) and other chronic pulmonary conditions (*n* = 22). Cardiac comorbidities included heart failure (*n* = 19), myocardial infarction (*n* = 9) PCI or CABG (*n* = 11) or previous heart valve replacement (*n* = 3); ****; Other diagnoses included: intoxications, anaphylaxis and gastro-intestinal bleed. After Bonferroni adjustment, a *p* value < 0.005 was regarded as significant

**Fig. 2:**
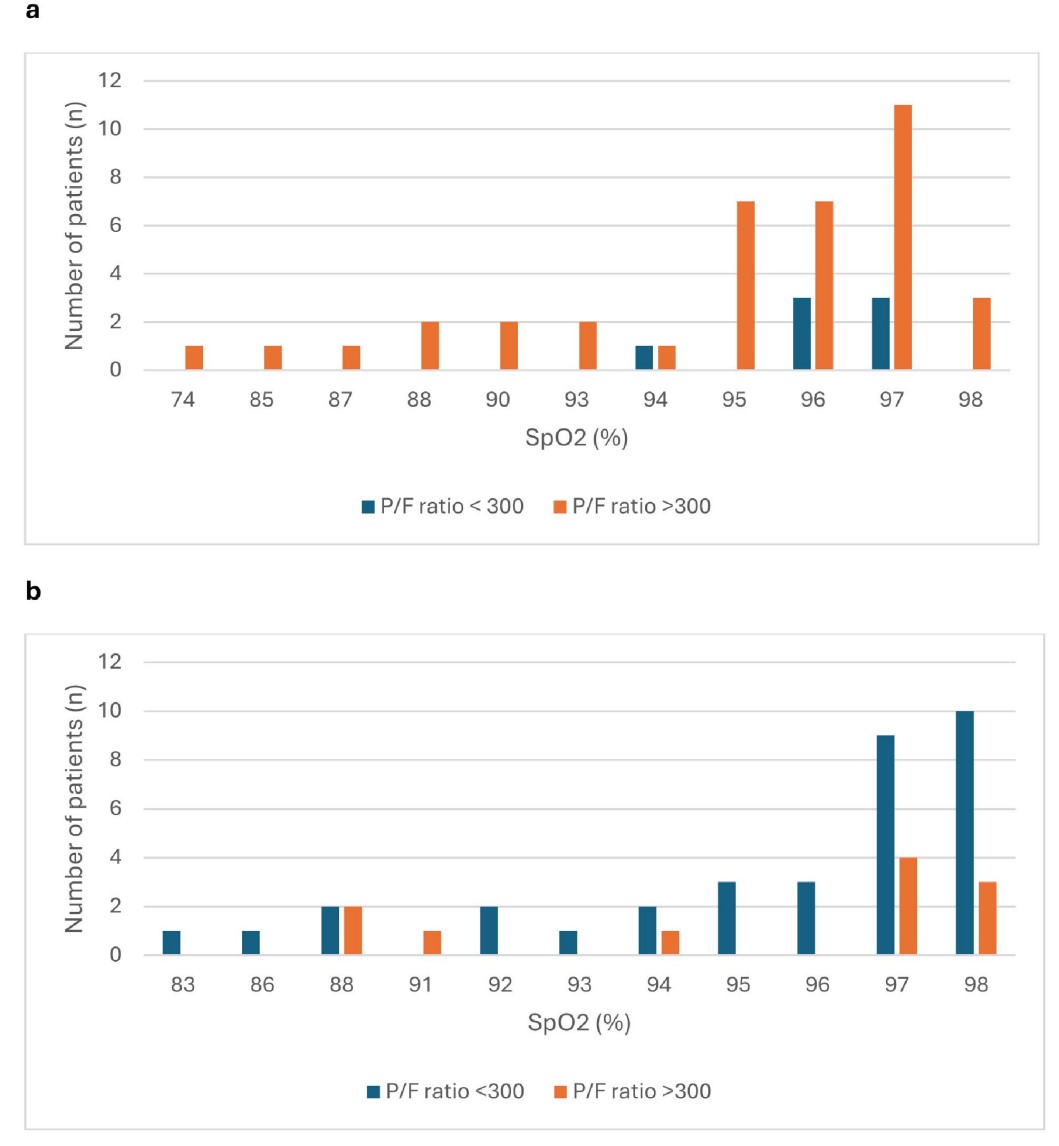
(**a**) Frequency distribution of SpO_2_ levels of patients with occult hyperoxia with *low* flow oxygen suppletion stratified by P/F ratio (n = 45); (**b**) Frequency distribution of SpO_2_ levels of patients with occult hyperoxia with *high* flow oxygen suppletion stratified by P/F ratio (n = 45)

Figure 2 shows the frequency distribution of SpO_2_ levels of patients with occult hyperoxia (SpO_2_ within or below target range, but PaO2 > 13,5 kPa) stratified by their P/F ratio. The vast majority of patients with occult hyperoxia receiving oxygen via a nasal canula (low flow) had an SpO_2_ between 95% and 97% and had a high P/F ratio (Fig. 2a). The 7 patients with a low P/F ratio were all within their SpO_2_ target range. On the contrary, the majority of patients receiving oxygen via a high flow device (ventimask or non-rebreathing mask) had saturation of 97 or 98% and all but 112 patients had a P/F ratio < 300, indicative of underlying ventilatory compromise (Fig. 2b).

## Discussion

In this cohort study, we found that when oxygen is titrated based on SpO_2_ levels, this results in occult hyperoxemia in a significant proportion of the patients. EMS personnel should especially be reluctant to administer (low flow) oxygen as a standard of care to patients who do not have clear respiratory compromise, as these patients are at a high risk of developing (occult) hyperoxia.

The potential harmful effects of long-term hyperoxia on vascular tone and cellular integrity are well known [4, 8]. Animal studies have indicated that even short-term (1-hour) hyperoxia maybe harmful, inducing functional and morphological changes in rat brains [21], and long-term changes in DNA-repair pathways, even at FiO2 levels as low as 0.3 [22]. In humans, brief exposure of only 15 min to a high FiO2 has been shown to result in an increase in systemic vascular resistance, with potential effects on CO [23, 24]. Although no studies have been published so far on the potential negative effects of (ultra) short exposure to moderate FiO2 levels (as most patients were exposed to in this study), based on these findings, hyperoxia should be avoided if possible.

Therefore several treatment guidelines nowadays focus on the prevention of both hypoxia- and hyperoxia [11, 12]. This study demonstrates however, that awareness of and/or adherence to these guidelines in the prehospital setting is not yet optimal: In 40% of the patients presented to the ED who demonstrated hyperoxia in their ABGA, SpO_2_ values were above the generally recommended target range of 94–98% (88–92% in severe COPD).

Interestingly, in patients with a history of ischemic heart disease, EMS providers seemed to be more aware of the potential risks of hyperoxia, as in this subgroup, obvious hyperoxia was less often present. This could be explained by the early and explicit emphasis placed by several societies on the potential deleterious effects of hyperoxia in patients with an acute coronary syndrome (ACS) or an out-of-hospital cardiac arrest [25, 26]. Interestingly, for patients with COPD we found the opposite: obvious hyperoxia (with SpO_2_ values above 92%) was encountered more often in this subgroup of patients, which may be a reflection of the generally higher concerns of developing hypoxia in this group.

This study demonstrates that in the majority of the cases, hyperoxia remains undetected by measuring SpO_2_ values alone. Over 60% of the patients in our cohort had occult hyperoxia: These patients had normal (or even decreased) SpO_2_ levels in the presence of an PaO2 > 13,5 Kpa. This may be explained by various reasons. First, the reliability of the SpO_2_ relies on the quality of the plethysmogram. In patients wearing nail polish, in shocked patients with cold extremities, in sick patients who are shivering or otherwise have movement artefacts, and in patients with dysrhythmia’s it is difficult to obtain a reliable SpO_2_ trace, and the SpO_2_ value represented may be an underestimation of actual values, resulting in unnecessary (high amounts of) oxygen administration More importantly, the relation between SpO_2_ and PaO_2_ is described by the oxygen dissociation curve. This means that in the upper range of saturations measured, a wide range in PaO_2_ values may be present: whereas some patients will be normoxemic, others will be hyperoxemic.

Although SpO_2_ is not a perfect tool to guide oxygen suppletion, often it is the only tool available, as it is important to start oxygen suppletion early to prevent hypoxia in many critically ill patients, and guidance of suppletion by arterial blood gasses (as is done in the intensive care unit (ICU)) is not always possible or desirable in the prehospital setting or the ED. Therefore it is important to establish how occult hyperoxia can be prevented when we only have SpO_2_ as a guidance, and our findings provide some clues:

In patients with occult hyperoxia, the majority (> 75%) of patients receiving *high* flow oxygen (by ventimask or NRM) had a P/F ratio < 300, indicative of ARDS [27, 28]. These patients are likely also clinically pulmonary compromised, and hence oxygen therapy is started liberally to prevent hypoxia. For further guidance and prevention of hypoxia, blood gas analysis is warranted. In these patients, a brief period of hyperoxia is likely unavoidable. In contrast, > 75% of the patients receiving *low* flow oxygen had a P/F ratio > 300. This shows that in patients who are likely clinically less compromised from a pulmonary perspective, and in whom oxygen is started (e.g. to meet increased metabolic demands or for comfort [29, 30]) through a nasal cannula, are at a particular high risk of developing hyperoxia. In these patients, who are generally also only moderately sick (judged by their average triage category), prehospital and ED clinicians should carefully weight potential risks and benefits of oxygen administration, and overall be more reluctant before starting oxygen.

### Limitations

This study had several limitations. First, as this was a proof-of-principle descriptive study, no sample size estimation was performed. Some trends may therefore have remained undetected or may have yielded non-significant results.

Second, patients were selected from the Acutelines data-, image and biobank of a single university hospital in the Netherlands. Only patients with the most-urgent NTS triage categories were included (as only these were included in the biobank). This may have affected the generalizability of our findings, although patients with the lowest triage categories rarely receive oxygen suppletion.

Further, FiO_2_ levels for calculation of P/F ratio’s were estimated (based on the method of oxygen delivery and the amount of oxygen given) and not measured. Although it is often assumed that the fraction of oxygen that is inspired (above the normal atmospheric level) increases by 4% for every additional liter of oxygen flow administered there may be inter- patient variability in the actual administered FiO2, depending on respiration rate, and pattern (mouth open or closed) [31]. Also, since no FiO_2_ values were noted in patients receiving non-invasive ventilation (NIV), no conclusion can be drawn about these patients concerning their P/F ratios.

Finally, as the primary outcome in our study was the number of patients with occult hyperoxia, and as we only included patients with confirmed hyperoxia in their ABGA, confounding by indications may be present. Therefore our findings should be interpreted with caution: Although our results demonstrate that hyperoxia remains undetected by SpO2 guided oxygen titration in a significant number of cases, no conclusions can be drawn regarding overall prevalence of hyperoxia in cohorts were SpO2 guided titration is used.

## Conclusion

When oxygen is titrated based on SpO_2_ levels, this results in occult hyperoxemia in a significant proportion of the patients. Healthcare providers should especially be reluctant to administer (low flow) oxygen as a standard of care to patients who do not have clear respiratory compromise, as these patients are at a high risk of developing (occult) hyperoxia.

## Data Availability

The dataset used and/or analysed during the current study are available from the corresponding author, after consulting with the board of Acutelines, on reasonable request.

## Abbreviations

ABGA: Arterial blood gas analysis
ACS: Acute coronary syndrome
ARDS: Acute respiratory distress syndrome
CABG: Coronary artery bypass grafting
CO: Cardiac output
COPD: Chronic obstructive pulmonary disease
CTc: Central ethical committee
ED: Emergency department
EMS: Emergency medical service
FiO_2_: Inspired oxygen fraction
ICU: Intensive care unit
NIV: Non-invasive ventilation
NRM: non rebreathing mask
NTS: Netherlands triage standard
O_2_: Oxygen
PaO_2_: Partial arterial oxygen pressure
PCI: Percutaneous coronary intervention
P/F: Partial arterial oxygen pressure / inspired oxygen fraction
ROS: Reactive oxygen species
SpO_2_: Peripheral capillary oxygen saturations
UMCG: University medical center Groningen
VM: Ventimask
